# A lifetime economic model of mortality and secondary care use for patients discharged from hospital following acute stroke

**DOI:** 10.1177/17474930241284447

**Published:** 2024-09-29

**Authors:** Peter McMeekin, Stephen McCarthy, Andrew McCarthy, Jennifer Porteous, Michael Allen, Anna Laws, Phil White, Martin James, Gary A Ford, Lisa Shaw, Christopher I Price

**Affiliations:** 1 Department of Nursing, Midwifery & Health, Northumbria University, Newcastle upon Tyne, UK; 2 University of Exeter Medical School, Exeter, UK; 3 Peninsula Applied Research Collaboration (PenARC), University of Exeter, Exeter, UK; 4 Translational and Clinical Research Institute, Newcastle University, Newcastle upon Tyne, UK; 5 Oxford University Hospitals NHS Foundation Trust and Medical Sciences Division, University of Oxford, Oxford, UK; 6 Population Health Sciences Institute, Newcastle University, Newcastle upon Tyne, UK

**Keywords:** Economics, stroke, cost factors, modeling, mortality, burden of disease

## Abstract

**Background:**

The long-term health-economic consequences of acute stroke are typically extrapolated from short-term outcomes observed in different studies, using models based on assumptions about longer-term morbidity and mortality. Inconsistency in these assumptions and the methods of extrapolation can create difficulties when comparing estimates of lifetime cost-effectiveness of stroke care interventions.

**Aims:**

To develop a long-term model consisting of a set of equations to estimate the lifetime effects of stroke care interventions to promote consistency in extrapolation of short-term outcomes.

**Methods:**

Data about further admissions and mortality were provided for acute stroke patients discharged between 2013 and 2014 from a large English service. This was combined with data from UK life tables to create a set of parametric equations in a model that use age, sex, and modified Rankin Scores to predict the lifetime risk of mortality and secondary care resource utilization including ED attendances, non-elective admissions, and elective admissions. A cohort of 1509 (male 51%; mean age 74) stroke patients had median follow-up of 7 years and represented 7111 post-discharge patient years. A logistic model estimated mortality within 12 months of discharge, and a Gompertz model was used over the remainder of the lifetime. Hospital attendances were modeled using a Weibull distribution. Non-elective and elective bed days were both modeled using a log-logistic distribution.

**Results:**

Mortality risk increased with age, dependency, and male sex. Although the overall pattern was similar for resource utilization, there were different variations according to dependency and gender for ED attendances and non-elective/elective admissions. For example, 65-year-old women with a mRS at discharge of 1 would gain an extra 6.75 life years compared to 65-year-old women with a mRS at discharge of 3. Over their lifetime, 65-year-old women with an mRS at discharge of 1 would experience 0.09 less ED attendances, 2.12 less non-elective bed days, and 1.28 additional elective bed days than 65-year-old women with an mRS at discharge of 3.

**Conclusions:**

Using long-term follow-up publicly available data from a large clinical cohort, this new model promotes standardized extrapolation of key outcomes over the life course and potentially can improve the real-world accuracy and comparison of long-term cost-effectiveness estimates for stroke care interventions.

**Data assess statement:**

Data are available upon reasonable request from third parties.

## Introduction

Studies into the effectiveness and cost-effectiveness of stroke care interventions mostly follow individuals for only a short time-frame, but the impact on health and resource utilization often persists across lifetimes. These longer-term consequences are typically estimated by extrapolating from short-term effects with standard assumptions about mortality and health events, which themselves may not reflect the whole life course. For example, a recent systematic review identified 21 economic evaluations of thrombectomy before 2019, including 15 with a long time horizon (beyond 20 years) but found a significant heterogeneity in modeled assumptions.^1^ Dichotomized measures of dependency are generally used during these projections without recognition of states in-between. Consequently, lifetime estimates of relative cost-effectiveness for stroke care interventions may be influenced as much by the underlying assumptions than the effect of the intervention itself and lack sufficient granularity to allow individual predictions based upon patient level data.

As there are no standard models available to extrapolate the health effects of stroke care over time, it can be difficult for clinicians and service planners to compare between interventions long-term. In other disease areas with high levels of comorbidity and recurrent events, notably diabetes, models have been developed that estimate lifetime effects for individual patients.^2^ To support comparison of stroke interventions and facilitate prediction of benefit, the European Stroke Organisation’s Health Economics Working Group has similarly recommended the “Development of resources for the standardisation of economic evaluation.”^3^ The aim of this study was to develop a long-term model to predict the lifetime effects of stroke care interventions based upon circumstances at discharge, using inputs typically applied during economic evaluation and data from median 7 years (ranging from 5.7 years to 8.3 years) of follow-up from a large, hospitalized stroke population in England. This model is an effectiveness model which can then be used in further cost-effectiveness analyses.

## Methods

### Model population

A cohort of 1509 patients discharged from a single large acute stroke unit in North-East England between January 1, 2013, and December 31, 2014, provided follow-up data until censor on April 2, 2021. The unit had 24/7 specialist physician input to decisions about emergency stroke treatment, which at the time included intravenous thrombolysis but without routine access to thrombectomy (this only generally being undertaken within the Pragmatic Ischemic Stroke Thrombectomy Evaluation trial). All patients received CT brain imaging and MRI was available to confirm a diagnosis of ischemic stroke if required. There were no exclusions for inpatient and outpatient multidisciplinary care, which followed national clinical guidelines including screening for and treatment of vascular risk factors such as hypercholesterolemia, hypertension, diabetes, atrial fibrillation, and carotid stenosis.^4^ Approximately a third of patients received inpatient rehabilitation for physical, language, and perceptual impairments, with access to community rehabilitation as required. The Early Supported Discharge service was available. Routine clinical care IT systems captured information for each patient about subsequent death, Emergency Department (ED) attendances (date of attendance(s)), non-elective (total length of stays, days), and elective (total length of stays, days) admissions. Only ED attendances, non-elective and elective admissions to the index hospital were included. It is possible that patients attended other hospitals within the United Kingdom, although this would affect a small number of patients. This means our estimates may be underestimates of the cohort’s true secondary care usage. It was assumed missing modified Rankin score (mRS) at discharge were missing at random and these patients were excluded from the analysis. Data was linked and anonymized by the care provider. No patient-identifiable data is reported. As a data modeling project comprising only routinely collected items and with no impact on patient care, NHS research ethics committee approval was not required. Approval for data transfer was granted from the hospital data controller.

### Model overview

The long-term model consists of a set of risk equations that estimate life expectancy and lifetime secondary care usage. Each equation takes mRS score at discharge, age at stroke onset, and sex as inputs to predict survival or resource use in order to calculate the risk of an event at a single point in time, the cumulative risk up to a point in time or the number of events up to a point in time. To estimate life expectancy, due to the higher risk of death in the first year, an equation was developed to estimate mortality within the first year of stroke and a separate equation was developed to estimate mortality in subsequent years. For secondary care usage, a separate equation was developed from the follow-up of the model population to predict resource usage for each key secondary care event: ED attendances, non-elective admissions, and elective admissions.

Simulation using Life tables was used to estimate the relative mortality risk of patients in our cohort compared to the general population.^5^ Lifetime secondary care use was estimated from the patient cohort adjusted for projected survival and was defined as ED attendances, elective bed days and non-elective bed days. This study was reported in accordance with the Strengthening the Reporting of Observational Studies in Epidemiology (STROBE) statement (and is available in the Supplemental Material).^6^

#### Statistical analyses

All but one of the equations in the model use hazard functions to reflect increasing risk of mortality and secondary care resource utilization. The exception is death within 12 months of discharge, which uses a logistic form. Each underlying hazard was examined graphically, and the most appropriate modeling approach was selected according to Akaike’s information criterion (AIC) from a choice of exponential, Weibull, logistic, log-logistic, and Gompertz parametric forms.^7,8^ Proportional hazard assumptions were tested by examination of Schoenfeld residuals in comparable Cox models.^9^ Illustrative outputs are presented using the base case (all regression coefficients set to zero), which is a female of mean (cohort) age and a mRS at discharge of 0. All analyses were carried out using Stata version 14.0 software and R.^10,11^

#### Prediction of mortality

The approach taken by the UK Diabetes Outcome mode was used for mortality prediction, whereby risk equations are based upon parametric relationships reflecting the non-monotonic nature of the underlying data.^2^ As there was a higher risk of mortality in the first year after stroke, which fell before increasing again in subsequent years, it was necessary to have two risk equations: a logistic prediction of death within 12 months of discharge, and a Gompertz form for survivors beyond the first year.

**Figure 1.**
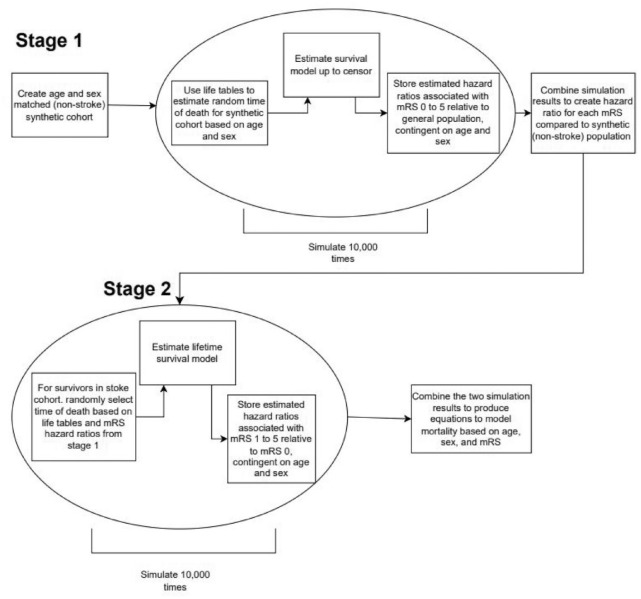
Diagram of long-term model to predict mortality beyond 12 months after stroke discharge.

The estimation of mortality beyond 12 months was conducted in two simulation stages as set out in Figure 1. The first stage required the creation of a synthetic patient control cohort without stroke based upon the age and sex distribution of the model stroke population. Life tables were used to estimate a random time of death for this control cohort based upon age and sex across 10,000 simulations.^12^ Within each simulation, a Gompertz model was developed by combining the stroke cohort with the synthetic control group. These models were based on age, sex and mRS with interactions between these variables explored and included where model fit was improved. The resulting coefficients from all simulations were then averaged to create a set of hazard ratios of death for each state captured by the mRS on discharge compared with the control cohort of non-stroke patients.

During the second-stage simulation, each stroke patient surviving at censor point was given a random future date of death, based on their age, sex, and hazard ratio associated with their mRS score at discharge (estimated during the first simulation stage). Lifespan was truncated at 100 years of age (due to life tables containing no data over 100 years) and the simulation was again run 10,000 times to estimate the lifetime Gompertz model. The additional risk of death associated with each disability state (at discharge following stroke), compared with the UK population (from life tables), was added to the risk of death for those stroke patients alive at censor point. The resulting probability of being alive at any point in time was equal to 1 minus the probability of death in year 1 multiplied by the probability of 1 minus the probability of death in that year.

#### Prediction of secondary care resource utilization

Survival functions were chosen for each of the three equations to represent increasing resource use over time, where each occurrence of resource use was modeled as a failure. These functions estimated the total number of failures (i.e. secondary care events) over the patient’s expected lifetime. The algorithms are suitable for use in both discrete-event and discrete-time models (see Supplemental Materials).

Prediction beyond the time interval of the available data was possible by extrapolating along the distributions of the risk models using Stata’s *streg* command. Unlike mortality, secondary care events can occur multiple times per patient across their lifespan, and so estimates of resource use are dependent upon choosing an underlying probability distribution (such as an exponential or Weibull distribution) that appropriately reflects the behavior between time and event of interest. If an inappropriate distribution is chosen, then estimates may be inaccurate and misleading. To ensure appropriate distributions were chosen, different probability distributions were compared to each other using the AIC (an estimator of prediction error) and to the underlying data itself. The distribution with the best fit for each of the cumulative resource uses was chosen. For each ED attendance, the cumulative count was increased on the day of the attendance unless it occurred at the same time as a non-elective admission. Elective and non-elective admissions were accounted for in terms of cumulative days of bed occupancy. For example, a non-elective admission lasting 15 days was modeled as 15 sequential bed days.

## Results

### Baseline characteristics

The median follow-up per patient was 2187 days post-discharge from hospital following the index stroke (including any inpatient rehabilitation) and, overall, this represents 7110 years of cohort follow-up. Mean age was 74 years, 51% were male, and 99% were white British (Table 1). At censor date (April 2, 2021), 44% were alive.

**Table 1.** Baseline characteristics at hospital discharge.

	Total (n = 1509)	mRS 0 (n = 139)	mRS 1 (n = 333)	mRS 2 (n = 366)	mRS 3 (n = 359)	mRS 4 (n = 268)	mRS 5 (n = 44)
Follow-up (Years)	7110.53	881.16	1988.46	1965.13	1457.13	774.16	44.49
Mean Age (Years) (SD)	73.69 (12.90)	67.15 (13.12)	68.53 (12.81)	72.57 (12.36)	77.17 (10.94)	79.03 (11.88)	81.84 (11.68)
Median Age (Years) (Interquartile range)	76 (66–83)	69 (58–77)	70 (60–79)	74 (65–81)	79 (72–85)	81 (74–87)	84 (74–90)
Male (%)	50.96	69.06	60.36	52.73	45.68	37.69	31.82
Pre-stroke mRS (mean)	0.94	0.13	0.41	0.74	1.34	1.88	2.61
Diabetes at original discharge (%)	15.77	12.95	14.11	19.67	15.60	14.55	13.64
Atrial fibrillation at original discharge (%)	14.38	7.19	12.31	12.57	16.99	18.66	20.45
Hypertension at original discharge (%)	47.18	41.01	48.65	45.63	49.03	48.88	43.18
Ischemic stroke as original admission (%)	90.08	95.61	95.00	93.71	85.27	80.43	86.23
Median NIHSS at original stroke admission (IQR)	3 (2–8)	2 (1–3)	2 (1–4)	3 (2–5)	5 (3–10)	10 (4–18)	17 (8–22)
Thrombolysis treatment for original stroke (%)	12.76	11.51	9.31	12.02	14.21	11.57	20.45
Alive at censor (%)	44.40	71.94	65.47	51.64	30.92	19.40	0
Observed Emergency Department Attendances (mean)	4.09	4.78	4.04	4.49	4.57	3.08	1.18
Observed Non-Elective Bed Days (mean)	23.24	15.41	17.19	26.13	32.90	20.80	5.68
Observed Elective Bed-days (mean)	1.20	1.23	1.43	1.57	0.99	0.74	0.73
At least one subsequent acute cerebrovascular disease non-elective admissions (n)	206	15	51	59	60	19	2

Ischemic strokes made up 90% of the cohort, with diabetes, atrial fibrillation, and hypertension being present at discharge in 16%, 15%, and 47%, respectively. With increasing discharge mRS, the percentage of males decreased while age and admission stroke severity increased. We observed 252 deaths within 12 months and 587 between month 13 and censor. There was a total of 6137 ED attendances, 4234 non-elective admissions (accounting for 35,063 bed days), and 1255 elective admissions (accounting for 1770 bed days). Details of the 20 most common primary diagnoses of non-elective admissions are described in Supplemental Table 1. Over the follow-up, there were 206 subsequent strokes recorded in 169 patients, representing 11.2% of the cohort.

### Prediction of mortality

**Figure 2.**
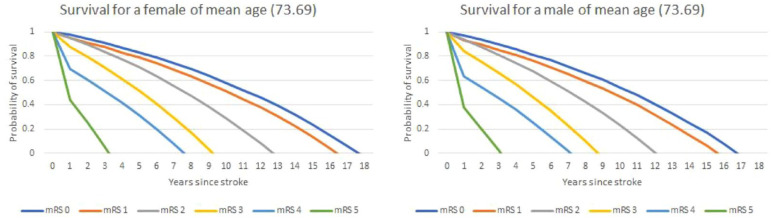
Survival curves for a patient of mean age (73.69).

The outputs of the two analyses to predict mortality post-discharge (logistic model for year 1 and Gompertz post year 1) are shown in Supplemental Table 2. The logistic model for year one mortality included only age at onset, sex, and discharge mRS as planned, whereas for overall lifetime mortality due to an interaction between age and mRS identified during the analysis, an age-squared variable and an interaction term with discharge mRS was included. The risk of mortality within 12 months increased with age and was greater for men. Greater (worse) discharge mRS was associated with a higher risk of death compared to those with a mRS of 0, although the higher risk with mRS of 1 (versus 0) was statistically insignificant. After 12 months, risk of death was greater for men and increased with age at an increasing rate for older patients. Greater mRS at discharge was associated with a higher risk of death, but this effect was not as powerful as increasing age at discharge. Figure 2 and Table 2 show the predicted lifetime survival following discharge for a patient of mean age for each possible discharge mRS 0 to 5.

**Table 2.** Sample Probabilities of Survival.

mRS	Sex	Probability of survival at the end of year 1	Probability of survival at the end of year 3	Probability of survival at the end of year 5	Probability of survival at the end of year 10	Median Life expectancy
0	Female	98%	91%	83%	58%	11.37
	Male	97%	90%	81%	54%	10.68
1	Female	95%	87%	79%	52%	10.24
	Male	93%	85%	76%	47%	9.53
2	Female	95%	84%	71%	29%	7.7
	Male	93%	81%	67%	23%	7.15
3	Female	87%	71%	51%	0%	5.12
	Male	84%	66%	46%	0%	4.64
4	Female	69%	52%	31%	0%	3.16
	Male	63%	45%	25%	0%	2.52
5	Female	44%	6%	0%	0%	0.85
	Male	38%	2%	0%	0%	0.71

### Prediction of secondary care resource utilization

ED attendances were best modeled using a Weibull distribution. Non-elective bed days and elective bed days were both best modeled using a log-logistic distribution. Sex and discharge mRS were included as categorical variables with age included as a continuous mean centered variable. The coefficients for the equations can be found in Supplemental Table 3. The models for secondary care use over the analysis period showed that ED attendances were lower for men than women (at any point in time) and increased with age. Compared to an individual with an original discharge mRS of 0, the same individual would have a lower estimated count of ED attendances if their mRS was 1 but a higher estimated count if their mRS was 2 or greater. For non-elective bed days, total count increased as age increased, with males having a lower number of bed days compared with females (all other variables being identical). The total number of non-elective bed days increased as the mRS increased, except for mRS of 5: this group had a higher total number of non-elective admission bed days than the same individual with an mRS of 0, 1, or 2 but a lower total bed days than if they had a mRS of 3 or 4. For the elective admission bed days, the total count increased with age but was greater for males. Compared to an individual with a mRS of 0, the same individual would have a higher elective admission bed day count if their mRS was 1, 2, 3, or 5 but a lower total bed day count if their mRS was 4.

### Model application

Using the coefficients and equations from Supplemental Tables 2 and 3, predictions can be made about the life expectancy of people discharged from hospital following a stroke based on their sex, age at time of stroke, and mRS at time of discharge. Table 3 shows the estimated median life expectancy (assumed to be the time when probability of being alive is 0.5) for a range of patient characteristics.

**Table 3.** Sample predictions.

Patient	Survival median years (IQR)	Lifetime secondary care resource use		
		Emergency department attendances	Non-elective bed days	Elective bed-days
Female 65, mRS 1	15.92 (9.41–19.60)	8.84	25.20	2.73
Female 65, mRS 3	9.17 (4.44–11.33)	8.93	27.32	1.45
Female 65, mRS 5	1.25 (0.54–1.40)	1.54	11.03	1.06
Male 65, mRS 1	15.01 (8.55–18.46)	7.90	23.00	2.75
Male 65, mRS 3	8.47 (3.78–10.32)	7.84	25.00	1.45
Male 65, mRS 5	1.05 (0.44–1.28)	1.26	8.15	0.99
Female 75, mRS 1	9.51 (4.91–11.88)	6.04	23.55	2.26
Female 75, mRS 3	4.67 (1.97–5.57)	5.35	24.68	0.97
Female 75, mRS 5	0.8 (0.33–1.28)	1.12	9.84	0.81
Male 75, mRS 1	8.83 (4.29–10.93)	5.33	21.26	2.27
Male 75, mRS 3	4.21 (1.60–4.91)	4.62	22.21	0.95
Male 75, mRS 5	0.67 (0.28–1.15)	0.91	6.91	0.74
Female 85, mRS 1	5.14 (2.36–6.27)	3.81	21.29	1.72
Female 85, mRS 3	2.31 (1.06–2.57)	3.14	21.88	0.58
Female 85, mRS 5	0.53 (0.22–1.05)	0.84	8.86	0.61
Male 85, mRS 1	4.7 (1.99–5.61)	3.32	18.89	1.71
Male 85, mRS 3	2.05 (0.86–2.25)	2.67	19.28	0.56
Male 85, mRS 5	0.46 (0.19–0.92)	0.69	6.11	0.58

By matching the estimates of life expectancy with the corresponding expected annual resource use for each combination of key characteristics, it is also possible to model the secondary resources consumed over the lifetime of a patient/cohort of patients. For example, Table 3 shows that a cohort of 65-year-old men discharged from hospital with an mRS of 1 would be expected to live (on average) 15.01 years and during this time would average 7.90 ED Attendances, 23.00 non-elective bed days and 2.75 elective bed days. In addition, it is possible to show the effect of improved mRS scores on patients. For example, 65-year-old women with a mRS at discharge of 1 would live an extra 6.75 years compared to 65-year-old women with a mRS at discharge of 3. Over their lifetime, 65-year-old women with an mRS at dsicharge of 1 would experience 0.09 less ED attendances and 2.12 less non-elective bed days but 1.28 additional elective bed days than 65-year-old women with an mRS at discharge of 3.

For consistency with previous economic evaluation models, which typically have used dichotomized independent (mRS 0-2) versus dependent (mRS 3-5) states as their outcomes, the Supplemental Material also includes mortality and resource use equations for these binary outcomes (Supplemental Tables 4–6).

## Discussion

It is common practice for health-economic evaluations to be extrapolated across patient lifetimes to reflect important differences in cost and outcomes, which cannot be determined through clinical trials without prolonged and expensive follow-up.^12^ As most stroke patients survive until discharge with a high burden of vascular illness and comorbidities, it is important that data-driven projections of survival and resource utilization can be made for planning care provision and understanding the likely impact of new interventions (via impact on discharge mRS, or indeed other reasonably early post-stroke mRS). Typically, this has been achieved by applying assumed relationships between population mortality and the additional mortality observed in the study population. While models based on these types of assumptions account for uncertainty by varying probabilities, they lack granularity due to the use of binary outcomes (e.g. independent versus dependent/dead), and their accuracy is influenced considerably by differences between the population where they are applied and that which was used to derive parameters for the longer-term assumptions. In addition, NICE have introduced a new proportionate approach to technology appraisals, which uses a single model to span treatment options within a disease area, avoiding the repetition of developing and reviewing models.^13^ Our model can be incorporated into such a pathway for stroke treatments, reflecting the cost and health benefits of improving stroke outcomes.

By using a large sample of patients undergoing standard stroke care and analysis approaches reflecting the distribution of data for key variables, our model can produce statistically robust estimates of life expectancy and secondary care resource use. These could be used in economic models for similar populations, for example, confirming that the longer-term benefits of treatments like thrombolysis/thrombectomy are as expected. A key strength of our risk equations is that they are derived from a cohort who were followed up for 7–8 years outside of a clinical trial, and therefore the longer-term outputs are far more likely to represent real-world settings.

Our predictions of mortality are similar to those of previous models.^14^ For example, a 50-year-old woman with mRS 0 in our model has an estimated survival of 31.18 years versus 32 years predicted by Shavelle et al.^14^ However, there are also potentially important differences. Shavelle et al. excluded from their synthesis patients who did not survive at least 3 months. In patient groups with a low 3-month mortality, the expected life expectancies are similar in both models. However, in patient groups with a high 3-month mortality (older patients with higher mRS), the results can be quite different. For example, a 70-year-old man with mRS 5 has an expected survival of 0.83 years in our model, compared to 5 years predicted by Shavelle et al. In addition, this might reflect that data from non-UK stroke survivors recruited between 1981 and 2009 was used to inform the Shavelle model, when there were major secular trends in emergency treatment, rehabilitation, and secondary prevention. Furthermore, their estimates of mortality were calculated by multiplying risk ratios against life tables, whereas we utilized an algorithm-driven approach.

Before survival is considered, it is unsurprising that advancing age is associated with greater resource utilization. However, the relationships between dependency and gender appear to be more complex. For elective admissions, the lowest number occurred in the middle mRS range (mRS 3–4), which is likely to reflect interventions among ambulant post-stroke individuals such as joint replacement surgery. It is unclear why there was a reduction in elective admissions for mRS 4 and an increase again for mRS 5. Assuming this is a genuine reduction, it may reflect a need for hospitalization of the patient to provide care that cannot be provided in the community because of pre-existing poor health (e.g. when immobility prohibits attendance for an outpatient procedure) or to offer palliation. It should, however, be acknowledged that the mRS data reflect the status of patients at discharge from the hospital following an index stroke, and their health may well have subsequently changed for both stroke and non-stroke reasons, creating noise within the resource utilization data. Sex differences for ED attendance/non-elective admissions (female higher) and elective admissions (male higher) after stroke have not previously been reported. These are likely to reflect a combination of factors including comorbidities and social support, but further exploration with a richer health and social care dataset than was available to this study is required to understand this observation.

It is possible to use the outcome and resource utilization equations to estimate the lifetime cost and quality-adjusted life years (QALYs) consequences of different mRS. The Supplemental Materials include illustrative examples based on assumed utilities and costs in pounds sterling per resource utilized (Supplemental Tables 7–9), which demonstrate how, at the typical NICE “willingness to pay” threshold of £20,000 per QALY, most improvements in mRS would be considered cost-effective even after the lifetime secondary care costs are considered.^12,15,16^ For example, the model predicts that if a 65-year-old woman were discharged with an mRS of 1 instead of 3, this would generate a net saving of £134,613 and discounted lifetime secondary care savings of £12,869, before any other costs that are not captured in the model are considered. An interactive online calculator that illustrates the use of our equations (including financial costs and QALYs) is available at https://stroke-predictions.streamlit.app/Lifetime_outcomes. In the Supplemental Materials and the online calculator, the costs used were £137 for an ED attendance, £444 for elective bed day and £533 for a non-elective bed day.

### Limitations

The model risk equations are based upon a set of patients from one English locality and one healthcare organization, who were predominantly of white British ethnicity. Although this limits the generalizability to the European or global stroke population, life expectancy and health status in the North-East of England for both sexes are significantly poorer than the average across England as a whole, possibly resulting in a more pessimistic outlook by modeled estimates.^17^ Lack of data about the additional risks of mortality associated with different mRS beyond our 8-year follow-up means there could be bias associated with applying an observed additional mortality risk. This is most likely to overestimate the risk of death in younger, lower mRS patients, many of whom would live beyond the censor point. In addition, it was assumed mRS was missing at random and those patients were excluded from the analysis, although this may not be the case.

The model reflects patients who were admitted to hospital with a specialist-confirmed diagnosis of stroke and were alive at the time of discharge. Not all patients would have been eligible for all treatment types, such as thrombolysis, and the admission period for the cohort means that thrombectomy was not routinely available. There will have been other changes in care introduced since the time when the cohort experienced an index event, which may also have an impact on discharge mRS, prevention of future cardiovascular events, and subsequent resource use. Future research should consider how to account for different treatment eligibility and introduction of new therapies in data used for mortality and resource predictions.

Although 90-day outcomes are common for many trials, the SSNAP dataset is collected at discharge and 6 months post-discharge.^18^ As there was limited data collected at 6 months, our model used only mRS at discharge, but there is evidence that mRS at discharge is stable up to at least 30 days, suggesting that this model will still be relevant.^19^ The standard and readily available set of predictors included in our model are limited in number; therefore, predictions may not be reliable regarding an individual patient’s prognosis. For example, certain clinical characteristics may affect the model such as hemorrhagic versus ischemic stroke, and patients with new atrial fibrillation started on anticoagulants. Studies such as Xu have suggested that hemorrhages are more costly than infarctions, although this may be mediated solely through greater disability states.^20^ However, the inclusion of additional predictors would limit the wider value of our equations, as this level of information would often be unavailable or not be as consistently reliable as age, sex, and discharge mRS. However, this limitation is far less of an issue when models are used in the lifetime projections for larger population subgroups where the focus is on the relative effects of changes in dependency.

Economic models taking wider perspectives should include information about primary, social, and informal care costs over the estimated life course. Currently, our models of resource use and associated costs only relate to secondary care due to the data availability. This represents a potential underestimate of the costs of stroke. However, it would be possible to extend the methods used in this model to estimate other resource categories, where data is available and more detailed work would be required to stratify the population into subgroups with different resource needs. External validation of the model is now required using more recent data from other settings, both in the United Kingdom and international context. An additional future aim should be to define a common set of input variables to enable comparison of models being used to extrapolate the clinical and economic effects of differing stroke care interventions.

## Conclusion

Using data from the long-term follow-up of a large cohort of English stroke patients, we have developed a model to estimate future mortality and secondary care resource utilization. This offers the possibility of increasing the consistency of economic modeling for examining the impact of changes in stroke care and reducing the risk of predictions being driven more by assumptions made during extrapolation of short-term outcomes. Further development and validation of the model could extend the perspective of future economic models.

## Supplemental Material

sj-docx-1-wso-10.1177_17474930241284447 – Supplemental material for A lifetime economic model of mortality and secondary care use for patients discharged from hospital following acute stroke Supplemental material, sj-docx-1-wso-10.1177_17474930241284447 for A lifetime economic model of mortality and secondary care use for patients discharged from hospital following acute stroke by Peter McMeekin, Stephen McCarthy, Andrew McCarthy, Jennifer Porteous, Michael Allen, Anna Laws, Phil White, Martin James, Gary A Ford, Lisa Shaw and Christopher I Price in International Journal of Stroke

## References

[1] Wu X Khunte M Gandhi D , et al. A systematic review of cost-effectiveness analyses on endovascular thrombectomy in ischemic stroke patients. Eur Radiol 2022; 32: 3757–3766. 35301558 10.1007/s00330-022-08671-0

[2] Hayes AJ Leal J Gray AM Holman RR Clarke PM. UKPDS outcomes model 2: a new version of a model to simulate lifetime health outcomes of patients with type 2 diabetes mellitus using data from the 30 year United Kingdom Prospective Diabetes Study: UKPDS 82. Diabetologia 2013; 56: 1925–1933. 23793713 10.1007/s00125-013-2940-y

[3] Cadilhac DA Kim J Wilson A , et al. Improving economic evaluations in stroke: a report from the ESO Health Economics Working Group. Eur Stroke J 2020; 5: 184–192. 32637652 10.1177/2396987319897466 PMC7313366

[4] National Clinical Guideline for Stroke. National Clinical Guideline for Stroke for the UK and Ireland, https://www.strokeguideline.org/ (2023, accessed 2 August 2024).

[5] Jackson C Stevens J Ren S , et al. Extrapolating survival from randomized trials using external data: a review of methods. Med Decis Making 2017; 37: 377–390. 27005519 10.1177/0272989X16639900 PMC5424081

[6] von Elm E Altman DG Egger M , et al. Strengthening the Reporting of Observational Studies in Epidemiology (STROBE) statement: guidelines for reporting observational studies. BMJ 2007; 335: 806–808. 17947786 10.1136/bmj.39335.541782.AD PMC2034723

[7] Akaike H. A new look at the statistical model identification. IEEE Trans Autom Control 1974; 19: 716–723.

[8] George B Seals S Aban I. Survival analysis and regression models. J Nucl Cardiol 2014; 21: 686–694. 24810431 10.1007/s12350-014-9908-2 PMC4111957

[9] Schoenfeld D. Partial residuals for the proportional hazards regression model. Biometrika 1982; 69: 239–241.

[10] StataCorp. Stata statistical software. Release 14. College Station, TX: StataCorp; 2015.

[11] R Core Team. R: a language and environment for statistical computing, https://www.R-project.org/ (2021, accessed 2 August 2024).

[12] National Institute for health and Care Excellence. NICE health technology evaluations: the manual, https://www.nice.org.uk/process/pmg36 (2023, accessed 2 August 2024).

[13] National Institute for health and Care Excellence. Taking a proportionate approach to technology appraisals, https://www.nice.org.uk/about/what-we-do/proportionate-approach-to-technology-appraisals#:~:text=The%20pathway%20approach%20uses%20a, the%20consistency%20of%20our%20recommendations (2022, accessed 2 August 2024).

[14] Shavelle RM Brooks JC Strauss DJ Turner-Stokes L. Life expectancy after stroke based on age, sex, and rankin grade of disability: a synthesis. J Stroke Cerebrovasc Dis 2019; 28: 104450. 31676160 10.1016/j.jstrokecerebrovasdis.2019.104450

[15] Dijkland SA Voormolen DC Venema E , et al. Utility-weighted modified rankin scale as primary outcome in stroke trials. Stroke 2018; 49: 965–971. 29535271 10.1161/STROKEAHA.117.020194 PMC5895119

[16] Mihaylova B Briggs A O’Hagan A , et al. Review of statistical methods for analysing healthcare resources and costs. Health Econ 2010; 20: 897–916. 20799344 10.1002/hec.1653 PMC3470917

[17] Office for National Statistics. Health state life expectancies in England, Northern Ireland and Wales, https://www.ons.gov.uk/peoplepopulationandcommunity/healthandsocialcare/healthandlifeexpectancies/ (2021, accessed 2 August 2024).

[18] ElHabr AK Katz JM Wang J , et al. Predicting 90-day modified Rankin Scale score with discharge information in acute ischaemic stroke patients following treatment. BMJ Neurol Open 2021; 3: e000177. 10.1136/bmjno-2021-000177 PMC8231000 34250487

[19] Quinn TJ Dawson J Walters MR Lees KR. Functional outcome measures in contemporary stroke trials. Int J Stroke 2009; 4: 200–205. 19659822 10.1111/j.1747-4949.2009.00271.x

[20] Xu XM Vestesson E Paley L , et al. The economic burden of stroke care in England, Wales and Northern Ireland: using a national stroke register to estimate and report patient-level health economic outcomes in stroke. Eur Stroke J 2018; 3: 82–91. 29900412 10.1177/2396987317746516 PMC5992739

