# Supplementary material for: A lifetime economic model of mortality and secondary care use for patients discharged from hospital following acute stroke
Source: Int J Stroke. 2024 Sep 29;20(1):116–25. doi: 10.1177/17474930241284447 (PMC11669260; doi:10.1177/17474930241284447)
Supplement: sj-docx-1-wso-10.1177_17474930241284447 – Supplemental material for A lifetime economic model of mortality and secondary care use for patients discharged from hospital following acute stroke [file sj-docx-1-wso-10.1177_17474930241284447.docx]

**Supplementary Materials**

**1. Most common primary diagnoses of non-elective admissions**

Supplementary Table 1: Most common primary diagnoses of non-elective admissions

| Primary Diagnoses | n | % |
| --- | --- | --- |
| Acute cerebrovascular disease | 1888 | 45.06% |
| Pneumonia (except that caused by tuberculosis or sexually transmitted disease) | 303 | 7.23% |
| Other connective tissue disease | 247 | 5.89% |
| Urinary tract infections | 209 | 4.99% |
| Nonspecific chest pain | 183 | 4.37% |
| Epilepsy, convulsions | 128 | 3.05% |
| Congestive heart failure; nonhypertensive | 120 | 2.86% |
| Chronic obstructive pulmonary disease and bronchiectasis | 116 | 2.77% |
| Cardiac dysrhythmias | 96 | 2.29% |
| Superficial injury, contusion | 94 | 2.24% |
| Other nervous system disorders | 91 | 2.17% |
| Acute bronchitis | 87 | 2.08% |
| Transient cerebral ischemia | 86 | 2.05% |
| Septicemia (except in labor) | 83 | 1.98% |
| Syncope | 82 | 1.96% |
| Senility & organic mental disorders | 80 | 1.91% |
| Fracture of neck of femur (hip) | 79 | 1.89% |
| Intestinal infection | 74 | 1.77% |
| Coronary atherosclerosis and other heart disease | 73 | 1.74% |
| Acute & unspecified renal failure | 71 | 1.69% |
| Total | 4190 | 100.00% |

**2. Mortality Prediction**

There was a higher risk of mortality in the first year which fell in subsequent years before increasing again, violating the parametric relationships which are strictly non-increasing or non-decreasing i.e., the probability of death either increases over time or decreases over time and the proportionality of hazards with respect to mRS, which suggested a Gompertz form. However, the nature of the hazard functions and non-proportionality over the first year meant that two risk equations for mortality were developed to take account of stroke patient status in different epochs (periods).

**2.1 Mortality Equations**

Form of Gompertz

$H_{t}=\frac{e^{\alpha+{\sum_{i=1}^{n} \beta_{i}X}_{i}}\cdot(e^{\gamma t}-1)}{\gamma}$ [1]

Form of Logistic

$Y_{1}=\frac{1}{1+e^{-(\alpha+{\sum_{i=1}^{n} \beta_{i}X}_{i})}}$ [2]

Where:

$Y_{1}$ is the probability of death in year 1 (day 365.25)

$H_{t}$ is the cumulative hazard at time t, where t > 365.25

$t$ is the time in days since discharge

$e^{\alpha+{\sum_{i=1}^{n} \beta_{i}X}_{i}}$ is the linear predictor

$\gamma$ is the gamma term from the Gompertz function

Predictions

The cumulative probability of death by time t (where t > 365) is

P(Death ≤ t) = $1-\left( 1-Y_{1} \right)\cdot\left( 1-H_{t} \right)$ [3]

The probability of death during period t and t+1 is estimated by^1^:

P(t ≤ Death ≤ t+1) = $1-e^{\left( 1-\left( 1-Y_{1} \right)\cdot\left( 1-H_{t} \right) \right)-\left( 1-\left( 1-Y_{1} \right)\cdot\left( 1-H_{t+1} \right) \right)}$ [4]

The method used to calculate a time of death depends on whether the probability for which a time of death is being calculated from is less than the probability of death in year one. If the probability is greater than the probability of death in year one, the time of death based on a probability of death after year one is derived from equation [1], where $p$ is the desired death in days is, where the linear predictor is [1]

P(Death after year 1 = p) = $\ln\left( (\gamma\cdot p\cdot e^{-(\alpha+{\sum_{i=1}^{n} \beta_{i}X}_{i}})+1 \right)\cdot\gamma^{-1}+365$ [5a]

In years

P(Death after year 1 = p) = $\ln\left( (\gamma\cdot p\cdot e^{\alpha+{\sum_{i=1}^{n} \beta_{i}X}_{i}})+1 \right)\cdot{(365*\gamma)}^{-1}+1$ [5b]

Because the probability of death in year 1 is less than the probability of interest, it is necessary to modify equation [5] to allow for the probability of death. p becomes p’ and is given by the equation:

$p^{'}= \left( \left( p-\frac{1}{1+e^{-(\alpha+{\sum_{i=1}^{n} \beta_{i}X}_{i})}} \right)\cdot\left( 1- \frac{1}{1+e^{-(\alpha+{\sum_{i=1}^{n} \beta_{i}X}_{i})}} \right)^{-1} \right)$ [6]

If the probability of death in year one is greater than the probability of interest, the time of death in days in year one is derived from equation [2] where $p_{0}$ is the probability of death in year one (from [2]), and the probability of death for which a time is being calculated is $p$. This method is taken from Decision Modelling for Health Economic Evaluation^2^.

P(Death = p) $=\ln\left( 1-p \right)\cdot{(365ln (1-p_{0}))}^{-1}$ [7]

Examples:

*Each example uses the coefficients to four decimal places from Supplementary Table 1. As such estimates may be affected by rounding. Full coefficients are reported in the supporting materials.*

For an 80-year-old woman discharged from hospital with a modified Rankin Score of two, her probability of death in the 12 months following discharge would be:

- The logistic linear estimator is -4.2354 + (80-72.8575) · 0.0663 + 1.2590 = -2.50285
- From [2] $Y_{1}=\frac{1}{1+e^{-(-2.50535)}}$ = **0.0757**

For the same woman, her probability of death by the end of year two would be:

- The Gompertz linear estimator is -9.3173+ [0.0533 · (80-73.7324)] + [0.0002 · (80^2^ - 73.7324^2^)] + [-0.0018 · (80-73.7324)] + 0.5379 = -8.2639
- From [1] $H_{365}=\frac{e^{-8.2639}\cdot(e^{0.0002\cdot365}-1)}{0.0002}$ = 0.0975
- From [3] $1-\left( 1-0.0757 \right)\cdot\left( 1-0.0975 \right)$ = **0.1658**

And her probability of death during year three would be:

- The Gompertz linear estimator as above is -8.2639
- From [1] $H_{730}=\frac{e^{-8.2639}\cdot(e^{0.0002\cdot(2*365)}-1)}{0.0002}$ = 0.2025
- From [3], the cumulative probability of death $1-\left( 1-0.0757 \right)\cdot\left( 1-0.2025 \right)$ = **0.2628**
- From [4] $1-e^{(0.1658-0.2628)}$ = **0.0924**

Similarly, a 66-year-old male discharged with a modified Rankin Score of three would have the following probability of death in the 12 months following discharge:

- The logistic linear estimator is -4.2354 + (66-76.4884) · 0.0663 + 2.4773 + 0.2636 = -2.1899
- From [2] $Y_{1}=\frac{1}{1+e^{-(-2.1910)}}$ = **0.1007**

And the probability of death by the end of two years since discharge:

- The Gompertz linear estimator is -9.3173 + [0.0533 · (66-73.7324)] + [0.0002 · (66^2^ - 73.7324^2^)] + [0.0059 · (66-73.7324)] + 1.0163+ 0.0874= -8.8875
- From [1] $H_{365}=\frac{e^{-8.8875}\cdot(e^{0.0002\cdot365}-1)}{0.0002}$ = 0.0523
- From [3] $1-\left( 1-0.1007 \right)\cdot\left( 1-0.0523 \right)$ = **0.1477**

And during year two

- From [4] and above $1-e^{(0.1007-0.1477)}$ = **0.0459**

And his probability of death during year three:

- The Gompertz linear estimator as above -8.8875
- From [1] $H_{730}=\frac{e^{-8.8875}\cdot(e^{0.0002\cdot(2*365)}-1)}{0.0002}$ = 0.1085
- From [3], the cumulative probability of death $1-\left( 1-0.1007 \right)\cdot\left( 1-0.1085 \right)$ = **0.1983**
- From [4] $1-e^{(0.1477-0.1983)}$ = **0.0493**

To estimate the median time that a 65-year-old man, discharged with a modified Rankin score of two will survive is:

- From [2] check if death more than 50% probability in year 1
- The logistic linear estimator is -4.2354 + (65-72.8575) · 0.0663 + 1.2590 + 0.2758= -3.2217
- From [2] $Y_{1}=\frac{1}{1+e^{-(-3.2217)}}$ = **0.0384** which is less than the modelled 0.5
- From [7] with *p* = 0.5
- $p^{'}= \left( \left( 0.5-0.0384 \right)\cdot\left( 1-0.0384 \right)^{-1} \right)$ = 0.4800
- $\mathrm{Using} p^{'}\mathrm{from}\left[ 6 \right] in place of p in [5a]$ with a Gompertz linear predictor of -9.3173 + [0.0533 · (65-73.7324)] + [0.0002 · (65^2^ - 73.7324^2^)] + [-0.0018 · (65-73.7324)] + 0.5379+ 0.0874= -9.3840
- $\left[ 5a \right]\ln\left( \left( 0.0002\cdot{0.4800\cdot e}^{-(-9.3840)} \right)+1 \right)\cdot{0.0002}^{-1}$ = 3809
- To this add 365 as median survival is after year on and divide by 365 to give survival in years
- 3809 + 365 = 4174. 4174/365.25 = **11.43 years**

For a 71-year-old woman, discharged with a modified Rankin score of five, to estimate at what point she will have a 50% chance of still being alive:

- Her probability of death in year one is:
- From [2] 0.5128 which is greater than 0.5 so use [7], with linear predictor:
- -4.2354 + [0.0663 $\cdot$ (71-80.91837)] + 4.9444 = 0.05128
- $\ln\left( 1-0.5 \right)\cdot{(ln (1-0.5128)/365)}^{-1}$ = 352 days or 0.96 years

To identify at what point in time there will be a 40% change of her being dead:

- $\ln\left( 1-0.4 \right)\cdot{(ln (1-0.5128)/365)}^{-1}$ = 259 days or 0.71 years

**2.2 Mortality Coefficients**

For both the year one logistic equation and the lifetime Gompertz equation, co-efficient values greater than 0 suggest a higher risk of mortality and values less than 0 suggest a lower risk of mortality compared to the base case (constant only – female with mRS 0 and mean age).

Supplementary Table 2: Survival Analyses coefficients

|  | Year One Logistic | | | Lifetime Gompertz Hazard after Year One^2^ | | |
| --- | --- | --- | --- | --- | --- | --- |
| Variable | Coef^3^ | SE | 95% CI | Coef^3^ | SE | 95% CI |
| Constant | -4.2354 | 0.6057 | -5.4226, -3.0483 | -9.3173 | 0.0047 | -9.3266, -9.3081 |
| Gamma | - | - | - | 0.0002 | 0 | 0.0002, 0.0002 |
| Age at onset ¹ | 0.0663 | 0.0082 | 0.0503, 0.0823 | 0.0533 | 0.0005 | 0.0523, 0.0544 |
| Age at onset squared ¹ | - | - | - | 0.0002 | 0 | 0.0002, 0.0002 |
| Male | 0.2758 | 0.1601 | -0.0389, 0.5904 | 0.0874 | 0.0012 | 0.085, 0.0897 |
| mrs 1 * Age at onset ¹ | - | - | - | -0.0044 | 0.0002 | -0.0048, -0.004 |
| mrs 2 * Age at onset ¹ | - | - | - | -0.0018 | 0.0002 | -0.0021, -0.0014 |
| mrs 3 * Age at onset ¹ | - | - | - | 0.0059 | 0.0002 | 0.0055, 0.0062 |
| mrs 4 * Age at onset ¹ | - | - | - | 0.0034 | 0.0002 | 0.0029, 0.0038 |
| mrs 5 * Age at onset ¹ | - | - | - | -0.1241 | 0.0002 | -0.1245, -0.1238 |
| mrs 1 | 0.9539 | 0.6403 | -0.3012, 2.2089 | 0.1235 | 0.0023 | 0.1189, 0.128 |
| mrs 2 | 1.2590 | 0.6240 | 0.0360, 2.4821 | 0.5379 | 0.0039 | 0.5302, 0.5456 |
| mrs 3 | 2.4773 | 0.6058 | 1.2900, 3.6646 | 1.0163 | 0.0059 | 1.0046, 1.0279 |
| mrs 4 | 3.7310 | 0.6079 | 1.2901 3.6646 | 1.2936 | 0.2401 | 0.5385, 2.1107 |
| mrs 5 | 4.9444 | 0.6795 | 3.6126, 6.2763 | 2.5018 | 0.2716 | 1.5967, 3.3756 |
| ¹ Age variables are mean centred. The Gompertz model mean is 73.7324 years. The logistic model uses centred group means on mRS. These are 67.09161, 67.98058, 72.85753, 76.48837, 78.56029, 80.91837 for mRS 0-5 respectively.  ^2^ Schoenfeld hazard p=0.65, suggesting proportional hazards  ^3^ Coefficient values greater than 0 suggest a higher risk of mortality and value less than 0 suggest a lower risk of mortality compared to the base case (constant only – female with mRS 0 and mean age). Examples on how to apply these equations are given in the supplementary material. | | | | | | |

**3. Resource use prediction**

**3.1 Non-elective and Elective bed days Equations**

The estimates of the non-elective and elective bed days uses a loglogistic survival model, where the expected number of bed days is:

$$Count\left[ t_{i} \right]=-ln\left\{ \left( 1+\left[ (e^{-(\sum_{i=0}^{n} \beta_{i}X_{i})})\times t_{i}) \right]^{1/\gamma} \right)^{-1} \right\}$$

Where,

t_i_ is the median survival

γ is the ancillary parameter

$\sum_{i=0}^{n} \beta_{i}X_{i}$is the linear predictor

Examples:

*Each example uses the coefficients to four decimal places from Supplementary Table 2. As such estimates may be affected by rounding. Full coefficients are reported in the supporting materials.*

For an 80-year-old woman discharged from hospital with a modified Rankin Score of two, her median survival is 4.69 years. To calculate her expected number of non-elective admission bed days, the linear predictor would be:

- -1.334+(-0.0248*7.1425)+(0.2966*0)+(-0.4900) = -2.0011 (to four decimal places)

Therefore, her count of non-elective bed days would be:

- $Count\left[ 4.69 \right]=-ln\left\{ \left( 1+\left[ (e^{-(-2.0011)})\times4.69) \right]^{1/0.1617} \right)^{-1} \right\}$

Which equals 21.9330 non-elective bed days (to four decimal places)

Similarly, a 65-year-old male discharged with a modified Ranking score of three would have a median survival of 7.40 years. To calculate his expected number of elective admission bed days, the linear predictor would be:

- 1.2735+(-0.0081*-10.4884)+(-0.0774*1)+(-0.1613) = 1.1198 (to four decimal places)

Therefore, his count of elective bed days would be:

- $Count\left[ 7.40 \right]=-ln\left\{ \left( 1+\left[ (e^{-1.1198})\times7.40) \right]^{1/0.8516} \right)^{-1} \right\}$

Which equals 1.3392 elective bed days (to four decimal places)

**3.2 ED Attendances Equations**

The estimates of the number of ED attendances use a Weibull accelerated failure-time survival model, where the expected number of ED attendances is:

$$Count\left[ t_{i} \right]=-ln\left\{ e^{-(t_{i}^{\gamma} \times e^{-[(\gamma)\times(\sum_{i=0}^{n} \beta_{i}X_{i})]})} \right\}$$

Where,

t_i_ is the median survival

γ is the ancillary parameter

$\sum_{i=0}^{n} \beta_{0}X_{0}$is the linear predictor

Examples:

*Each example uses the coefficients to four decimal places from Supplementary Table 2. As such estimates may be affected by rounding. Full coefficients are reported in the supporting materials.*

For a 65-year-old male discharged with a modified Ranking score of two, his median survival time is 11.16 years. To calculate his expected number ED attendances , the linear predictor is:

- -0.0692+(-0.0050*-7.8575)+(0.0791*1)+(-0.1126) = -0.0634 to four decimal places.

Therefore, his expected count of ED attendances is:

- $Count\left[ 11.16 \right]=-ln\left\{ e^{-({11.16}^{0.8167} \times e^{-[\left( 0.8167 \right)\times\left( -0.0634 \right)]})} \right\}$

Which gives an expected count of ED attendances of 7.5530 (to four decimal places)

**3.3 Secondary Care Resource Utilisation Coefficients**

Compared to the base case (constant only – female with mRS 0 and mean age), a positive coefficient in all three equations leads to a decrease in the ED attendances, NEL bed days and EL bed days, while a negative coefficient leads to an increase.

Supplementary Table 3 Secondary care resource use hazard models

| **Variables** | **Emergency Department attendances (Weibull)** | | | **Non-EL Days (log-logistic)** | | | **EL Days (log-logistic)** | | |
| --- | --- | --- | --- | --- | --- | --- | --- | --- | --- |
|  | Coeff | SE | 95%CI | Coeff | SE | 95%CI | Coeff | SE | 95%CI |
| Constant | -0.0692 | 0.0601 | -0.187, 0.0487 | -1.3340 | 0.0329 | -1.3985,  -1.2695 | 1.2735 | 0.1167 | 1.0448, 1.5023 |
| Age ^1^ | -0.0050 | 0.0014 | -0.0076,  -0.0023 | -0.0248 | 0.0008 | -0.0264,  -0.0233 | -0.0081 | 0.0032 | -0.0144,  -0.0019 |
| Male | 0.0791 | 0.0331 | 0.0141, 0.1442 | 0.2966 | 0.0198 | 0.2577,  0.3354 | -0.0774 | 0.0720 | -0.2184, 0.0637 |
| mRS 1 | 0.1534 | 0.0614 | 0.0330, 0.2738 | -0.0468 | 0.0383 | -0.1218,  0.0283 | -0.7988 | 0.1273 | -1.0484,  -0.5492 |
| mRS 2 | -0.1126 | 0.0595 | -0.2291, 0.004 | -0.4899 | 0.0356 | -0.5598,  -0.4201 | -0.8763 | 0.1288 | -1.1287,  -0.6239 |
| mRS 3 | -0.4520 | 0.0597 | -0.5691,  -0.3349 | -1.1523 | 0.0358 | -1.2225,  -1.0821 | -0.1613 | 0.1331 | -0.4221, 0.0995 |
| mRS 4 | -0.3794 | 0.0674 | -0.5115,  -0.2474 | -1.2986 | 0.0386 | -1.3743,  -1.2229 | 0.1961 | 0.1586 | -0.1147, 0.5069 |
| mRS 5 | -0.3181 | 0.1808 | -0.6724, 0.0362 | -0.6244 | 0.0738 | -0.7689,  -0.4798 | -1.7254 | 0.2908 | -2.2953,  -1.1555 |
| ancillary parameter | 0.8167 |  |  | 0.1617 |  |  | 0.8516 |  |  |
| ¹ Age variables are mean centred. The hazard functions model mean is 73.7324 years. | | | | | | | | | |

**4. Dichotomous Model**

Supplementary Table 4: Survival Analyses for dichotomous

|  | Year One Logistic | | | Gompertz Hazard | | |
| --- | --- | --- | --- | --- | --- | --- |
| Variable | Coef | SE | 95% CI | Coef | SE | 95%CI |
| Constant | -3.1728 | 0.1934 | -3.5519, -2.7937 | -8.7208 | 0.0015 | -8.7237, -8.718 |
| Gamma | - | - | - | 0.0002 | 0 | 0.0002, 0.0002 |
| Age at onset ¹ | 0.1344 | 0.1531 | -0.1657, 0.4346 | -0.0134 | 0.0003 | -0.0139, -0.0128 |
| Age at onset squared ¹ | - | - | - | 0.0007 | 0.00E+00 | 0.0007, 0.0007 |
| Male | 0.2758 | 0.1601 | -0.0389, 0.5904 | 0.0945 | 0.0011 | 0.0923, 0.0967 |
| Dependent * Age at onset ¹ | - | - | - | 0.0027 | 0.0001 | 0.0025, 0.003 |
| Dependant | 2.2029 | 0.1825 | 1.8451, 2.5607 | 0.3514 | 0.0022 | 0.3471, 0.3557 |
| ¹ Age variables are mean centred. The Gompertz model mean is 73.7324 years. The logistic model uses centred group means on independent/dependant. These are 70.06683 and 78.21609 respectively | | | | | | |

Supplementary Table 5: Resource use survival models for dichotomous

| **Variables** | **Emergency Department attendances (Weibull)** | | | **NEL Days (log-logistic)** | | | **EL Days (log-logistic)** | | |
| --- | --- | --- | --- | --- | --- | --- | --- | --- | --- |
|  | Coeff | SE | 95%CI | Coeff | SE | 95%CI | Coeff | SE | 95%CI |
| Constant | -0.0642 | 0.0363 | -0.1354, 0.0069 | -1.6111 | 0.0157 | -1.6419, -1.5803 | 0.5616 | 0.0614 | 0.4412, 0.6821 |
| Age ^1^ | -0.005 | 0.0014 | -0.0076, -0.0023 | -0.0249 | 0.0008 | -0.0264, -0.0235 | -0.0071 | 0.0032 | -0.0135, -0.0008 |
| Male | 0.0858 | 0.033 | 0.0211, 0.1504 | 0.2938 | 0.0193 | 0.2559, 0.3316 | -0.093 | 0.0731 | -0.2362, 0.0502 |
| Dependant | -0.4334 | 0.0334 | -0.4988, -0.3679 | -0.9156 | 0.0194 | -0.9536, -0.8776 | 0.6038 | 0.0791 | 0.4487, 0.759 |
| ancillary parameter | 0.8161 |  |  | 0.1625 |  |  | 0.8695 |  |  |
| ¹ Age variables are mean centred. The hazard functions model mean is 73.7324 years. | | | | | | | | | |

Supplementary Table 6: Sample predictions for dichotomous

| **Patient at discharge** | **Survival Median (IQR)** | **Lifetime Secondary Resource Use** | | |
| --- | --- | --- | --- | --- |
|  |  | **Emergency Department attendances** | **Non-Elective bed days** | **Elective bed-days** |
| Female 65, independent | 12.23 (6.91 to 15.52) | 7.97 | 24.55 | 2.30 |
| Female 65, dependent | 8.88 (3.29 to 10.58) | 8.45 | 26.96 | 1.36 |
| Male 65, independent | 11.44 (6.36 to 14.53) | 7.03 | 22.33 | 2.33 |
| Male 65, dependent | 8.14 (2.79 to 9.63) | 7.34 | 24.62 | 1.37 |
| Female 75, independent | 6.92 (3.57 to 8.80) | 5.21 | 22.58 | 1.80 |
| Female 75, dependent | 4.33 (1.14 to 4.93) | 4.90 | 24.07 | 0.86 |
| Male 75, independent | 6.42 (3.26 to 8.12) | 4.57 | 20.31 | 1.82 |
| Male 75, dependent | 3.88 (0.96 to 4.39) | 4.17 | 21.59 | 0.85 |
| Female 85, independent | 3.48 (1.78 to 4.22) | 3.10 | 19.89 | 1.25 |
| Female 85, dependent | 1.8 (0.61 to 2.09) | 2.50 | 20.22 | 0.43 |
| Male 85, independent | 3.23 (1.64 to 3.86) | 2.72 | 17.62 | 1.26 |
| Male 85, dependent | 1.57 (0.55 to 1.89) | 2.08 | 17.58 | 0.42 |

**5. Example Cost-Utility modelling**

It is possible to use the equations to estimate the lifetime consequences of improvements or deterioration in mRS. We do this using the following financial costs £137 for an ED attendance, £444 for elective bad day and £553 for a non-elective bed day. We also assume (based on our data) that a portion of patients are discharged to long term nursing care and 95% of these people remain there for the rest of their lives (Supplementary Table 6). The cost of an ED attendance is calculated as an average of all ‘consultant led’ and ‘non consultant led’ A&E admissions from NHS Reference Costs and does not include transport costs due to lack of data availability^3^. The cost an elective bed day was taken from the literature and inflated to 2021 prices^4, 5^. The cost for a non-elective bed day was assumed to be the cost of an elective bed day with a 20% premium. The cost of a residential day was taken from Unit Costs of Health and Social Care 2021 where an average of the weekly cost of a ‘for profit’ and ‘not for profit’ residential care home for an older person was calculated^5^. Utilities to derive QALYS from life expectancy, age and mRS was calculated from the literature^6^ and standardized around the average age for each mRS cohort^6, 7^, reducing as each patients aged^8, 9^.

Supplementary Table 7: Portion of patients by mRS/ dichotomous that are discharged to long term nursing care

| Score | Percentage discharged to nursing care (%) |
| --- | --- |
| mRS 0 | 0.794 |
| mRS 1 | 0.984 |
| mRS 2 | 0.915 |
| mRS 3 (under 70) | 5.556 |
| mRS 3 (over 70) | 11.429 |
| mRS 4 (under 70) | 30.612 |
| mRS 4 (over 70) | 49.474 |
| mRS 5 (under 70) | 28.571 |
| mRS 5 (over 70) | 70.588 |
| Independent | 0.922 |
| Dependent | 14.094 |

Supplementary Table 8: Illustrative Estimated QALY Gains and Financial Consequences of improved mRS

| Patient | Old mRS | New mRS | Additional quality adjusted life years | Change in Resource Use (£) | Net Benefit (£) |
| --- | --- | --- | --- | --- | --- |
| Female, 65 | 1 | 0 | 1.08 | £501 | £22,018 |
| Female, 65 | 3 | 1 | 6.09 | £12,869 | £134,613 |
| Female, 65 | 5 | 3 | 5.03 | £931 | £88,304 |
| Male, 65 | 1 | 0 | 1.12 | £457 | £22,885 |
| Male, 65 | 3 | 1 | 6.05 | £11,877 | £132,896 |
| Male, 65 | 5 | 3 | 4.87 | -£1,170 | £83,853 |
| Female, 75 | 1 | 0 | 0.78 | £271 | £15,839 |
| Female, 75 | 3 | 1 | 4.49 | £15,465 | £105,249 |
| Female, 75 | 5 | 3 | 2.60 | £10,049 | £45,962 |
| Male, 75 | 1 | 0 | 0.82 | £210 | £16,603 |
| Male, 75 | 3 | 1 | 4.38 | £13,888 | £101,568 |
| Male, 75 | 5 | 3 | 2.45 | £6,538 | £41,019 |
| Female, 85 | 1 | 0 | 0.47 | £102 | £9,523 |
| Female, 85 | 3 | 1 | 2.68 | £7,530 | £61,175 |
| Female, 85 | 5 | 3 | 1.24 | £5,002 | £21,785 |
| Male, 85 | 1 | 0 | 0.52 | £8 | £10,345 |
| Male, 85 | 3 | 1 | 2.56 | £6,525 | £57,701 |
| Male, 85 | 5 | 3 | 1.14 | £3,042 | £18,876 |
| Net Benefit Calculated at £20,000 per QALY and future costs and QALYS are discounted at 3.5%  Negative Changes in Resource Use are extra costs to secondary care providers | | | | | |

A 75-year-old male who previously was discharged with an mRS of 1, now achieves an mRS 0 because of enhanced treatments gains, after discounting, 1.12 QALYs and incurs an extra £134 of secondary care use over their extended life span. This equates to a Net Benefit of £22,257 when QALYS are valued at £20,000. An 65-year-old woman, whose mRs moves from 3 to 1 sees a Net Benefit of £135,976, made up of 6.23 QALYs and £11,370 of reduced future secondary care use.

The same assumptions can be applied to dichotomous outcome data.

Supplementary Table 9: Illustrative Estimated QALY Gains and Financial Consequences of moving from dependant to independent.

| Patient | Additional quality adjusted life years | Change in Resource Use (£) | Net Benefit  (£) |
| --- | --- | --- | --- |
| Female, 65 | 5.00 | £37,081 | £137,036 |
| Male, 65 | 4.86 | £34,358 | £131,558 |
| Female, 75 | 3.34 | £33,142 | £100,001 |
| Male, 75 | 3.23 | £29,770 | £94,444 |
| Female, 85 | 1.88 | £13,801 | £51,497 |
| Male, 85 | 1.83 | £11,785 | £48,408 |
| Net Benefit Calculated at £20,000 per QALY and future costs and QALYS are discounted at 3.5%  Negative Changes in Resource Use are extra costs to secondary care providers | | | |

**6. STROBE Checklist**

STROBE Statement—checklist of items that should be included in reports of observational studies

|  | Item No | Recommendation | Page  No |
| --- | --- | --- | --- |
| **Title and abstract** | 1 | (*a*) Indicate the study’s design with a commonly used term in the title or the abstract | 1 |
|  |  | (*b*) Provide in the abstract an informative and balanced summary of what was done and what was found | 2 |
| Introduction | | | |
| Background/rationale | 2 | Explain the scientific background and rationale for the investigation being reported | 3 |
| Objectives | 3 | State specific objectives, including any prespecified hypotheses | 3 |
| Methods | | | |
| Study design | 4 | Present key elements of study design early in the paper | 3/4 |
| Setting | 5 | Describe the setting, locations, and relevant dates, including periods of recruitment, exposure, follow-up, and data collection | 3 |
| Participants | 6 | (*a*) *Cohort study*—Give the eligibility criteria, and the sources and methods of selection of participants. Describe methods of follow-up  *Case-control study*—Give the eligibility criteria, and the sources and methods of case ascertainment and control selection. Give the rationale for the choice of cases and controls  *Cross-sectional study*—Give the eligibility criteria, and the sources and methods of selection of participants | 3 |
|  |  | (*b*) *Cohort study*—For matched studies, give matching criteria and number of exposed and unexposed  *Case-control study*—For matched studies, give matching criteria and the number of controls per case | N/A |
| Variables | 7 | Clearly define all outcomes, exposures, predictors, potential confounders, and effect modifiers. Give diagnostic criteria, if applicable | 3 |
| Data sources/ measurement | 8* | For each variable of interest, give sources of data and details of methods of assessment (measurement). Describe comparability of assessment methods if there is more than one group | *3* |
| Bias | 9 | Describe any efforts to address potential sources of bias | 12 |
| Study size | 10 | Explain how the study size was arrived at | 3 |
| Quantitative variables | 11 | Explain how quantitative variables were handled in the analyses. If applicable, describe which groupings were chosen and why | 4-6 |
| Statistical methods | 12 | (*a*) Describe all statistical methods, including those used to control for confounding | 4-6 |
|  |  | (*b*) Describe any methods used to examine subgroups and interactions | N/A |
|  |  | (*c*) Explain how missing data were addressed | N/A |
|  |  | (*d*) *Cohort study*—If applicable, explain how loss to follow-up was addressed  *Case-control study*—If applicable, explain how matching of cases and controls was addressed  *Cross-sectional study*—If applicable, describe analytical methods taking account of sampling strategy | N/A |
|  |  | (*e*) Describe any sensitivity analyses | N/A |

Continued on next page

| Results | | | |
| --- | --- | --- | --- |
| Participants | 13* | (a) Report numbers of individuals at each stage of study—eg numbers potentially eligible, examined for eligibility, confirmed eligible, included in the study, completing follow-up, and analysed | N/A |
|  |  | (b) Give reasons for non-participation at each stage | N/A |
|  |  | (c) Consider use of a flow diagram | N/A |
| Descriptive data | 14* | (a) Give characteristics of study participants (eg demographic, clinical, social) and information on exposures and potential confounders | 6 |
|  |  | (b) Indicate number of participants with missing data for each variable of interest | N/A |
|  |  | (c) *Cohort study*—Summarise follow-up time (eg, average and total amount) | 6 |
| Outcome data | 15* | *Cohort study*—Report numbers of outcome events or summary measures over time | *7* |
|  |  | *Case-control study—*Report numbers in each exposure category, or summary measures of exposure |  |
|  |  | *Cross-sectional study—*Report numbers of outcome events or summary measures |  |
| Main results | 16 | (*a*) Give unadjusted estimates and, if applicable, confounder-adjusted estimates and their precision (eg, 95% confidence interval). Make clear which confounders were adjusted for and why they were included | 8 and supplementary material |
|  |  | (*b*) Report category boundaries when continuous variables were categorized | N/A |
|  |  | (*c*) If relevant, consider translating estimates of relative risk into absolute risk for a meaningful time period | supplementary material |
| Other analyses | 17 | Report other analyses done—eg analyses of subgroups and interactions, and sensitivity analyses | 6-9 |
| Discussion | | | |
| Key results | 18 | Summarise key results with reference to study objectives | 10 |
| Limitations | 19 | Discuss limitations of the study, taking into account sources of potential bias or imprecision. Discuss both direction and magnitude of any potential bias | 10-12 |
| Interpretation | 20 | Give a cautious overall interpretation of results considering objectives, limitations, multiplicity of analyses, results from similar studies, and other relevant evidence | 11-12 |
| Generalisability | 21 | Discuss the generalisability (external validity) of the study results | 10-12 |
| Other information | | | |
| Funding | 22 | Give the source of funding and the role of the funders for the present study and, if applicable, for the original study on which the present article is based | 12 |

**7. References**

1. Hayes AJ, Leal J, Gray AM, Holman RR and Clarke PM. UKPDS outcomes model 2: a new version of a model to simulate lifetime health outcomes of patients with type 2 diabetes mellitus using data from the 30 year United Kingdom Prospective Diabetes Study: UKPDS 82. *Diabetologia*. 2013; 56: 1925-33.

2. Briggs A, Sculpher M and Claxton K. *Decision Modelling for Health Economic Evaluation*. Oxford University Press, 2006.

3. Care DoHaS. NHS Reference Costs 2020 to 2021. London: Department of Health and Social Care, 2021.

4. Excellence NIfHaC. Perioperative care in adults. London National Institute for Health and Care Excellence, 2019.

5. Jones KCB, Amanda. *Unit Costs of Health and Social Care 2021*. University of Kent, Canterbury: Personal Social Services Research Unit, 2021.

6. Dijkland SA, Voormolen DC, Venema E, et al. Utility-Weighted Modified Rankin Scale as Primary Outcome in Stroke Trials. *Stroke*. 2018; 49: 965-71.

7. Mihaylova B, Briggs A, O'Hagan A and Thompson SG. Review of statistical methods for analysing healthcare resources and costs. *Health Econ*. 2011; 20: 897-916.

8. Ara R and Brazier JE. Populating an economic model with health state utility values: moving toward better practice. *Value Health*. 2010; 13: 509-18.

9. M Hernández Alava SP, A Wailoo Estimating EQ-5D by Age and Sex for the UK. Nice DSU, 2022.
